# Melt-Spun Fibers for Textile Applications

**DOI:** 10.3390/ma13194298

**Published:** 2020-09-26

**Authors:** Rudolf Hufenus, Yurong Yan, Martin Dauner, Takeshi Kikutani

**Affiliations:** 1 Laboratory for Advanced Fibers, Empa, Swiss Federal Laboratories for Materials Science and Technology, Lerchenfeldstrasse 5, CH-9014 St. Gallen, Switzerland; 2 Key Lab Guangdong High Property & Functional Polymer Materials, Department of Polymer Materials and Engineering, South China University of Technology, No. 381 Wushan Road, Tianhe, Guangzhou 510640, China; yryan@scut.edu.cn; 3 German Institutes of Textile and Fiber Research, Körschtalstraße 26, D-73770 Denkendorf, Germany; martin.dauner@ditf.de; 4 Tokyo Institute of Technology, 4259-J3-142, Nagatsuta-cho, Midori-ku, Yokohama, Kanagawa 226-8503, Japan; kikutani.t.aa@m.titech.ac.jp

**Keywords:** man-made fibers, bicomponent fibers, melt-spinning, fiber formation, spinnability, orientation, instabilities, technical textiles

## Abstract

Textiles have a very long history, but they are far from becoming outdated. They gain new importance in technical applications, and man-made fibers are at the center of this ongoing innovation. The development of high-tech textiles relies on enhancements of fiber raw materials and processing techniques. Today, melt spinning of polymers is the most commonly used method for manufacturing commercial fibers, due to the simplicity of the production line, high spinning velocities, low production cost and environmental friendliness. Topics covered in this review are established and novel polymers, additives and processes used in melt spinning. In addition, fundamental questions regarding fiber morphologies, structure-property relationships, as well as flow and draw instabilities are addressed. Multicomponent melt-spinning, where several functionalities can be combined in one fiber, is also discussed. Finally, textile applications and melt-spun fiber specialties are presented, which emphasize how ongoing research efforts keep the high value of fibers and textiles alive.

## 1. Introduction

Man-made fibers have a long history. Robert Hooke first brought up the idea to create silk-like fibers in 1665, followed by René-Antoine Ferchault de Réaumur, who actually produced the first artificial filaments from different kinds of varnish in 1734 [1]. In 1883 Joseph Swan injected dissolved nitro-cellulose into a coagulation bath and thus obtained filaments for light bulbs [2]. In 1938 DuPont de Nemours (Wilmington, DE, USA) launched the production of Nylon^®^ (PA 6.6), the first commercial melt-spun fiber, invented by Wallace Carothers [3]. In the same year Paul Schlack developed Perlon^®^ (PA 6), a fiber declared vital to the war by Nazi Germany [4]. The first polyester fiber, Terylene^®^ (PET), was created in 1941 by Imperial Chemical Industries (ICI) [5]. The commercial production of polyolefin fibers started in 1957, based on the Ziegler-Natta catalyst recognized by a Nobel Prize in 1963 [6].

Today, chemical fibers are spun by drawing a melt or solution of a polymer or an inorganic material from a spinneret into a medium (quenching or solvent removal by air/gas, water or coagulation bath) where it solidifies. Drawing can either be applied by godets (rollers) and winders, by a high-velocity air stream, or by an electrostatic or centrifugal force. Table 1 lists fiber spinning methods used to produce filaments, staple fibers and nonwovens.

In the last 80 years melt-spun fibers became by far the most important fibers for apparel, but even more so for technical textiles, where they spawned a myriad of novel applications. The aim of this review is to provide information about the current state of research and development regarding melt-spun fibers. The field has shown steady and continuous progress, and it is high time to summarize the technology from a contemporary point of view. This review provides insights in thermoplastic polymers as well as extrusion and bicomponent technology, with a strong focus on the main markets for melt-spun fibers.

## 2. Raw Materials for Melt-Spinning

### 2.1. Polymers and Their Spinnability

Most commonly used materials for melt-spinning are polyamides, polyesters and (linear) polyolefins [7]. Table 2 lists a selection of polymers used for fiber melt-spinning, together with some relevant properties. Basic requirement for melt-spinning is that the polymer becomes fusible below its degradation temperature. Note that the maximum allowed extrusion temperature can fall well below the decomposition temperature T_d_ quoted in Table 2, while some polymers have their melt-processing window near T_d_. In addition, such thermoplastic polymers should ideally have the following properties to ease processability and to yield sufficient fiber properties [8,9,10]:

- withstand extrusion temperature and shear strain at minimal degradation and without crosslinking (thermal stability);
- have sufficiently high molecular weight and thus enough melt-strength to prevent filament break under draw-down strain (too high molecular weight and thus too high viscosity can hamper processability);
- exhibit small polydispersity (narrow molecular weight distribution) to ensure consistent melt flow rheology (constant flow);
- have high enough mobility of the molecular chains to disentangle and unfold under stress and to orient in fiber direction under strain (linear polymers are most suitable);
- show high uniformity and purity to prevent fluctuations and blockage in processing.

The polydispersity of commercially available polymers ranges from two to 12 or more; as a rule of thumb, the polydispersity should not exceed three for stable melt-spinning [11]. Temperature, moisture, air humidity, residence time and shear forces significantly promote molecular weight degradation during extrusion and spinning. Local shear heating may increase the spinning temperature by as much as 10–15 °C [12].

Moisture can strongly influence processability and cause degradation of polymers in extrusion, thus drying of polymers is very important before extrusion [8]. This is especially true for polyesters like PET, PBT or PLA, which can suffer considerable loss in molecular weight by hydrolytic degradation (hydrolysis) of the melt in presence of water [13]. For water removal, either a batch procedure in fluidized bed or vacuum (tumble) dryers can be applied, or continuous drying in the feed hopper with desiccated air or nitrogen at normal pressure. However, over-drying polyamide by more than two orders of magnitude below the equilibrium moisture content can negatively influence extrusion processing, because moisture impacts the chemical equilibrium of the polycondensate and acts as a plasticizer for polyamide [14]. Non-hygroscopic polymers like polyolefins usually need not to be dried before processing. However, even hydrophobic fluoropolymers like PVDF should be dried to remove surface moisture, which otherwise could dissolve hydrogen fluoride (HF) monomers to form the highly corrosive and toxic hydrofluoric acid.

Polymers for man-made fibers can not only contain residual water, but also dissolved and dispersed gases, as well as volatile liquids and solids (e.g., unreacted monomers, reaction by-products) that boil at processing temperatures [15]. During extrusion of the polymer, these substances are kept in the melt by hydrostatic pressure. As their solubility decreases with the pressure drop at the die exit, gas bubbles and/or a pitted surface can evolve in the melt strand, which impair fiber quality or hinder spinnability [16]. Volatiles evaporating from the spinneret must be removed by an exhaust, both to protect operators and to avoid agglomeration at the die exit.

By far most of the man-made fibers are spun from semi-crystalline polymers. The crystalline structure stabilizes the highly orientated molecular chains, which otherwise tend to recoil above T_g_, resulting in pronounced fiber shrinkage [17]. In consequence, mainly amorphous polymers with high T_g_, like PEI and PC, are used for fiber melt-spinning (Table 2).

### 2.2. Polyamides

Globally, PA 6 and PA 6.6 are by far the most used polyamides that are also significant for large-scale production of melt-spun fibers [29]. PA 6.6 is produced by the condensation reaction of hexamethylenediamine and adipic acid, while PA 6 is synthesized by ring-opening polymerization of ε-caprolactam [30]. Both fiber types exhibit similar properties, i.e., outstanding wear and abrasion resistance, high tenacity and toughness, excellent fatigue behavior and good resilience (Table 2); slight dissimilarities mainly stem from differences in molecular weight distribution and draw-induced molecular orientation [18]. For industrial applications, fibers are drawn with DR 4–5 to achieve high mechanical performance, while DR 2-2.5 is applied for apparel applications to achieve high uniformity in dye diffusion [18]. Other well-tried fiber-forming aliphatic polyamides are PA 11 (T_m_~185 °C), PA 12 (T_m_~180 °C), PA 6.12 (T_m_~210 °C), PA 6.10 (T_m_~215 °C), PA 4 (T_m_~260 °C), PA 4.6 (T_m_~295 °C). In the nomenclature PA x.y, x and y represent the respective number of carbon atoms in the diamine and diacid monomer, respectively [31].

PA 5.6, which can be synthesized by direct polycondensation of 1,5-pentamethylenediamine obtained from L-lysine and adipic acid, is a biobased alternative to PA 6 and PA 6.6, with a high potential in fiber applications [32]. With T_m_~250 °C, PA 5.6 shows thermal properties and a heat resistance comparable to the commercially available PA 6 [33]. The melt-spinning of PA 5.6 in the form of segmented pie bicomponent fibers has been reported in combination with PET [34].

Some partially aromatic polyamides like polyphthalamides (e.g., PA 6T, T_m_~325 °C, and PA 9T, T_m_~300 °C) or PA MXD6 (T_m_~237 °C, produced from m-xylenediamine and adipic acid) can also be melt-spun; respective filaments are commercially available but scarce [35,36]. Aromatic polyamides (aramids), on the other hand, cannot be melt-processed, since their melting temperature exceeds the degradation temperature; aramid fibers are produced by solution spinning.

### 2.3. Polyesters

PET is the predominant polyester used for fiber production, not only because of its good end-use properties and economy of production but in particular because of the ease of physical and chemical modification, suppressing negative and enhancing positive properties of PET [37]. Due to its relatively high glass transition temperature (T_g_~75 °C), as-spun PET forms a stable, supercooled melt with molecular orientation in fiber direction, which develops oriented crystallites only when fully drawn [19]. Their excellent properties (Table 2) are responsible for polyester fibers and filaments finding use in all fields of fiber application [19]. To obtain higher molecular weight PET for improved performance, solid phase polymerization is applied below T_m_, where pre-crystallized chips are heated in a stream of hot inert gas or agitated in a vacuum drier to remove small traces of volatiles [37].

Other commercially viable polyesters suitable for fiber production are PBT and PTT, which exceed PET in crystallization rate, resilience, elasticity and dyeability (they can be dyed at 100 °C, while PET requires 130 °C) [22,37]. 1,3-propanediol, the crucial substrate to polymerize PTT (T_m_~230 °C), can either be derived petrochemically, or by enzymatic fermentation of renewable resources [38]. PEN, the last of this melt-spinnable semi-aromatic polyester family, has higher melt temperature (T_m_~270 °C), tensile modulus, chemical and UV-resistance than PET and as such is beneficial for industrial fibers [22,39]. Respective high performance melt-spun fibers are commercially available but scarce [40].

### 2.4. Polyolefins

The most prominent polyolefins used for melt-spinning are PP, LDPE and HDPE, consisting essentially of saturated aliphatic hydrocarbon macromolecules [41]. Technologies to convert polyolefins into fibers and fabrics include monofilament and multifilament spinning, staple fiber, spunbond, melt blown, and slit film [20]. Polyolefin-based spunbond and melt blown fabrics are the material of choice for disposable hygiene and medical applications like diapers, incontinence pants, sanitary napkins, surgical gowns and masks [42]. Polyolefin filaments, being polymeric hydrocarbons, possess luster and a waxy handle, which can be reduced by non-circular fiber cross-sections like triangular or cross-shaped [41]. The main properties and characteristics of polyolefin fibers are summarized in Table 2. Worth mentioning is their lightness (density below 1 g/cm^3^), poor dyeability and adhesion, as well as low resilience and high tendency to creep [20]. UHMWPE yields fibers with extraordinary tensile properties, but its very high molar mass hinders melt-spinning; the polymer needs to be gelled in a solvent before being extruded through a spinneret (gel-spinning) [43]. Toyobo (Manufacturer, Tokyo, Japan) introduced a melt-spun high-strength polyethylene fiber under the brand name Tsunooga^®^ [44].

### 2.5. Chemically Inert Polymers

Filaments from chemically resistant polymers like fluoropolymers, polyetherketones, polysulfides and polyetherimide find applications in e.g., hot medium filters or protective textiles [45]. The best-known fluoropolymer is PTFE (T_m_~330 °C), but its high molecular weight hinders flowability to an extent that it cannot be melt-spun [46]. Respective fibers are produced by paste extrusion, where PTFE powder is mixed with a lubricant and transformed into film to be calendered, slit, sintered and stretched [45]. A melt-processable PTFE material (T_m_~315 °C), comprising small amounts of perfluoropropylvinylether, was launched in 2006 under the brand name Moldflon^®^ (ElringKlinger Kunststofftechnik, Bietigheim-Bissingen, Germany) [47]. PVDF (T_m_~170 °C), PVF (T_m_~200 °C), and co-polymers of tetrafluoroethylene with e.g., hexafluoropropylene, have lower melting points and can be melt-spun to filaments with good tensile properties and high chemical resistance [24,27].

The melt-spinnable PEEK (T_m_~335 °C) is the foremost member of the aromatic thermoplastic polyetherketones [24]. The advantage of PEEK fibers is their ability to operate in extreme conditions (high temperature, chemical impact and abrasion) over long lifetimes [45]. PPS (T_m_~285 °C) is inherently flame-resistant, has outstanding high-temperature stability and oil and solvent resistance (no known solvent below 200 °C) [15,26]. PEI (T_g_~215 °C) is an amorphous polymer and can be melt-spun into fibers which are resistant against specific chemicals, have a lower strength and melting point than PEEK or PPS and are more extensible [24].

LCPs, characterized by a highly ordered fluid state, are resistant to virtually all chemicals [48]. The principal monomer in all commercial thermotropic LCPs is hydroxybenzoic acid [49]. By subjecting a LCP to shear and extension forces via melt-spinning, the molecular chains become highly oriented without post-drawing [50]. Subsequent heat-treatment (annealing) for solid phase polymerization under reduced pressure results in filaments with superior tensile properties [51,52]. The first commercially available, melt-spun LCP fiber was introduced in 1990 under the brand name Vectran^®^, now manufactured by Kuraray (Osaka, Japan) [53].

### 2.6. Thermoplastic Elastomers

Elastomeric fibers, produced from polymers with thermoreversible physical cross-links, are characterized by high elastic recovery (up to 99%) and high extensibility (up to 500%) [54,55]. Their elasticity performance mainly stems from the combination of soft and hard segments of the polymer structure [56]. Most conventional elastomeric fibers are produced by dry spinning of PU, but melt-spun products are available; although they often lack in yarn uniformity and recovery, they are of interest due to ecological and economic advantages [57]. In filaments, melt-spun from TPU, their outstanding elasticity originates from the chemical composition of polyols (soft segments) and isocyanates (hard segments) [58]. PEE, a multiblock copolymer comprising segments of semicrystalline polyester (hard, mostly PBT) and noncrystalline polyether (soft), is a low-cost melt-spinnable polymer with adequate characteristics as thermoplastic elastomer [54,59]. TPOs are melt-spun to produce elastic filaments that are chemically resistant [56,60]. Elastic recovery, heat-resistance and long-term stability of thermoplastic elastomers can be achieved by subsequent covalent crosslinking [59,61].

### 2.7. Amorphous Polymers

The morphology of solid-state polymers typically fluctuates continuously between ideal crystalline and fully amorphous states, where “amorphous” is widely used in polymer science to mean non-crystalline [62,63]. Polymers with irregular molecular structures cannot crystallize under any condition, so the only important morphological feature that can be changed through processing is molecular orientation [64]. X-ray diffraction studies have confirmed the presence of oriented non-crystalline domains in filaments, melt-spun from fully amorphous polymers, indicating that the degree of orientation is directly proportional to the fiber draw ratio [65,66].

Fully amorphous polymers tend to be transparent, in contrast to semicrystalline polymers, which typically are translucent or opaque due to a usually heterogeneous crystalline structure that leads to refractive index inhomogeneities and thus light scattering at the interfaces between crystalline and amorphous regions [67,68]. Most prominent examples of polymers used for melt-spinning transparent fibers are PMMA (T_g_~110 °C), PC (T_g_~150 °C) and PS (T_g_~95 °C) [26,69]. The application temperature of respective fibers is below T_g_, since their oriented macromolecules tend to recoil above T_g_, resulting in strong shrinkage of the fibers.

### 2.8. Biopolymers

The term “biopolymer” generally refers to biobased (produced from biogenic substances which are considered renewable resources), but is every so often used for biodegradable (degradable by biological means), biocompatible (no adverse effect on humans or animals) or bioresorbable polymers (dissolved or absorbed in the body). The main biopolymers considered for fiber melt-spinning are PLA, PCL, PGA, PBAT, PEF and PHAs.

PLA is produced from lactic acid, whose raw material is naturally occurring starch, which is usually extracted from corn [23]. Fiber grade PLA, mostly consisting of L lactic acid (LLA) containing less or equal to 8% D lactic acid (DLA), is commercially one of the most promising bio-based, biodegradable and biocompatible polymers (PLA with a D-isomer level exceeding 8% does not crystallize) [21,23,70]. The biodegradability of PLA in the natural environment is lower than that of other biopolymers, since it is less susceptible to microbial attack [71]. Its main drawback regarding melt-processing is the low thermal stability in the presence of moisture (hydrolysis) [72]. However, mechanical performance and thermal resistance can be enhanced by adjusted LLA/DLA mixing and adequate spinning parameters to obtain stereo-complex crystals with strong interaction between LLA and DLA sequences [28,73,74,75].

PCL (T_m_~60 °C) is a petroleum-based biodegradable and biocompatible aliphatic polyester with good mechanical properties, consisting of a sequence of methylene units with in-between ester groups [76]. Its slow biodegradation rate in the human body make PCL suitable for implantable long-term drug delivery systems [77]. PCL is highly miscible and combines well with other polymers, and thus has been investigated as a polymer blend component or copolymer for various applications [78,79,80,81]. PCL in the form of filaments is also of interest for technical textiles, but its low melting point limits the application and thus only a few studies discuss the conventional melt-spinning of PCL homo-component fibers [82,83,84,85].

PGA (T_m_~225 °C), a highly biocompatible and biodegradable petroleum derived aliphatic polyester of simple molecular structure, can be melt-spun in fibers with good mechanical properties [77,86]. Since the product placement of the first man-made absorbable suture named Dexon^®^ in 1972, PGA and its copolymers dominate the biodegradable suture market [87,88]. However, the narrow processing window makes it difficult to spin high-strength fibers under ordinary industrial conditions, while laboratory trials with modifications in the spinning line led to fibers of high tensile strength and toughness [89,90].

PBAT (T_m_~120 °C) is an aliphatic-aromatic copolyester, which degrades within a few weeks with the aid of naturally occurring enzymes [91]. Considering both tensile and biodegradation properties, a random copolymer with 44 mol% of polybutylene terephthalate was found to be ideal [92]. PBAT shows good melt-spinnability, and the copolymer’s hard (aromatic) and soft (aliphatic) monomer segments yield elastic fibers with low modulus and high resilience [93]. The copolyester is also considered a good candidate for the enhancement of PLA [91].

PEF (T_m_~210 °C) is produced by polycondensation of ethylene glycol and furan dicarboxylic acid (as such chemically analogous to PET), which can be derived from plant-based resources [94]. Despite its slow crystallization and low T_m_, PEF has recently gained attention as a potential bio-based replacement for PET [95]. In analogy to PET, solid phase polymerization is the key to attain high molecular weight and thus suitability for engineering applications [95]. Own unpublished data show that PEF can be melt-spun into fibers with properties similar to PET fibers.

PHAs are produced by bacteria for intracellular carbon and energy storage [96,97,98,99,100]. PHAs, either in the form of homopolymers or copolymers of various hydroxyalkanoic acids, are thermoplastic, biodegradable, biocompatible and nontoxic [101,102,103]. The bacteria-synthesized, perfectly linear and isotactic polymer chains promise superior properties [104,105,106]. However, their rapid thermal degradation at temperatures just above the melting temperature, variations in quality and molecular weight, and lack of purity commonly resulting from the biotechnical production process, hamper melt-spinning of virgin PHAs [107]. Melt-spinning trials have been reported for P3HB (T_m_~180 °C) [107,108,109,110,111,112], PHBV (T_m_~170 °C) [113,114,115] and PHBH (T_m_~145 °C) [116,117,118,119]. In 2007, Tepha (Lexington, KY, USA) launched P4HB (T_m_~60 °C) mono- and multifilaments for medical applications [120,121].

## 3. Additives for Melt-Spinning

### 3.1. Function of Additives

Additives are considered to facilitate processing, to provide additional functions, or to improve properties and durability of the final fiber (Table 3). The additive should exhibit thermal stability and good processability. As additives also can lead to blockage and fluctuations in processing, their quantity and variety should be kept as low as possible. The maximum tolerable amount depends on miscibility in the case of polymers, and solubility in the case of liquids.

Solid additives (powders) should exhibit uniformity, dispersibility, particle and aggregation size below 5% of the fiber diameter, good wettability with the matrix polymer, and they should not be prone to clogging. As a rule of thumb, to maintain processability, the amount of solid additives should not exceed 5%. Depending on additive and filament type, far less might be acceptable to avoid impairing spinnability and mechanical properties. Additives can also cause problems like degradation of the polymer, unwanted chemical reactions and gas liberation during processing. In the final filament, the presence of foreign matter can cause failure by cavitation, as well as fibrillation at the polymer-additive interface.

The benefit of additives turns into a challenge when degradability or recyclability of the polymers are required. Additives in biodegradable polymers must also be degradable and ecologically friendly, which limits availability. Widely used inorganic fillers like TiO_2_ can cause problems when released to the environment in substantial quantity. Upon recycling, residual additives that lost their original function are incorporated in the recycled mixed materials and impair targeted properties. The success of PET bottle flakes in fiber production mainly stems from the absence of colorants, which would turn fibers from recycled PET grey.

### 3.2. Processing Aids

The drawback of melt-spinning, that the polymer can oxidatively decompose during processing, can be counteracted by antioxidants that suppress radical chain reactions until consumed; Antioxidants are for example used to enable melt-blowing of polypropylene spunbond waste [122]. Hydrolytic degradation of polyesters can be mitigated by stabilizers that react preferably with water and/or carboxylic groups and thus affect the reaction kinetics of hydrolysis [123]. Nucleating agents like talcum powder or boron nitride are used to improve the onset of crystallization with the result that crystals become smaller and more homogenous, leading to better mechanical properties of spun filaments [124]. Dispersive additives can act as lubricants for the matrix polymer by reducing apparent viscosity, and thus temperature or pressure required for extrusion [125]. In consequence, lubricants can even render polymers melt-spinnable whose melting temperature exceeds their degradation temperature [126]. Polymer processing aids like fluoropolymers can delay the onset of flow instabilities by thinly coating the die wall, thus reducing adhesion between polymer and wall and in consequence promote slippage [127,128].

### 3.3. Enhancing Additives

Stabilizers improve longevity of polymers by suppressing degradation resulting from UV radiation and other high-energy sources. Hindered phenols and hindered amine light stabilizers (HALS) deactivate existing radicals, while UV absorbers dissipate the harmful radiation as less harmful heat [129]. Flame retardant additives can act either in the gas phase by quenching radicals in the flame and/or in the condensed phase by catalyzing the formation of a protective char layer [130]. They can also work synergistically with other polymer additives (light stabilizers, surfactants, fillers etc.) [131]. Plasticizers are blended with polymers to improve their flexibility and elasticity [132]. On the other hand, fillers can constrain the mobility of polymer chains, resulting in higher stiffness and resistance to creep [133,134].

### 3.4. Functional Additives

Dyes and pigments are used to impart color; while dyes are solubilized in the polymer matrix, pigments are polymer insoluble, non-migrating additives. Dope-dyed filaments are produced by adding inorganic and organic pigments in the form of colorant masterbatches to the matrix polymer during extrusion. These masterbatches often contain further additives like UV-stabilizers. To reduce gloss of man-made fibers, TiO_2_ or other metal oxides are added as delustrants. In order to melt-spin intrinsically antistatic fibers, mainly fillers like carbon black, carbon nanotubes, graphene or metal powders are used as electrically conductive additives [135,136,137]. Adding metal and metal oxide nanoparticles directly into the polymer matrix is a common approach to render man-made fibers antimicrobial [138]. Typically, the mechanical properties of man-made fibers change significantly when fillers are used; hence, bicomponent fibers with a functional compound as minor element are an interesting approach [34]. Examples are conductive fibers as described in paragraph 7.5, or the translucent fiber Bodyshell^®^ (Toray, Tokyo, Japan) with star-shaped pigmented core, which prevents white swim suits from losing their opaqueness in wet condition [139].

## 4. Polymer Melt-Spinning

### 4.1. Melt-Spinning Technique

**Figure 1:**
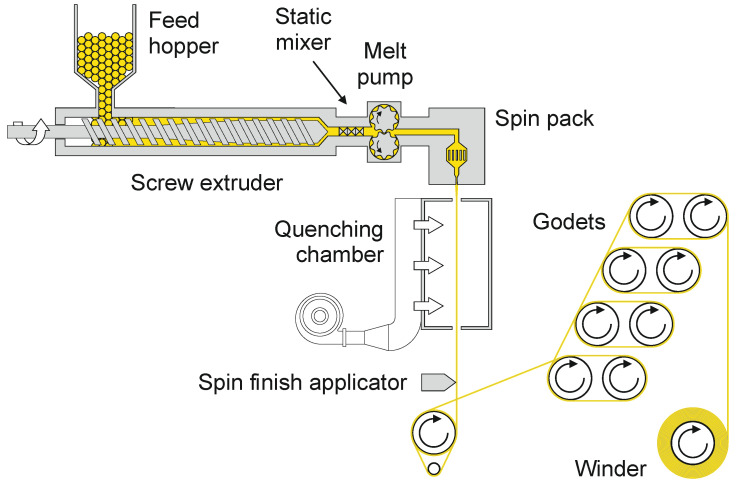
Schematic assembly of a melt-spinning line. By way of illustration, the polymer is represented in yellow.

A typical melt-spinning line comprises a screw extruder, a spin pack and a filament draw-down unit (Figure 1). Polymer pellets, granulates or chips are fed from the hopper to a single screw extruder to be melted and pressurized. In addition, a side extruder (not shown in Figure 1) can feed masterbatches, mainly applied for dope-dyed yarns. A melt pump ensures a defined and accurate throughput rate. The spin pack comprises polymer filtering and distribution parts, as well as the spinneret that is responsible for the filament formation. The overall design of the extrusion and spinning line must avoid melt stagnation by abrupt tapering or dead spots, where the polymer can degrade locally and discharge intermittently into the melt stream.

Leaving the spinneret, the extruded strands are either spun into a quenching chamber or a water bath for solidification. After cooling and application of a spin finish, the filaments are drawn (online or offline) by several godets. To improve drawability, the godets are heated, or the filaments can be guided over hot plates or through stretching ovens. Finally, a winder is used to spool the filaments on a bobbin.

### 4.2. Extrusion Line

In most cases, polymers in the form of pellets are melted in a screw extruder by applying heat and shear. Additives for coloring or to impart other properties are usually added with a carrier resin (masterbatch). The use of powders and liquids sometimes cannot be prevented, but it makes processability very challenging. The role of the extruder is not only to melt, but also to homogenize the polymeric material and to build-up the primary pressure needed for an effective operation of the melt pump. In large-scale applications, the melt is fed to the spinning system directly from the polycondensation line.

**Figure 2:**
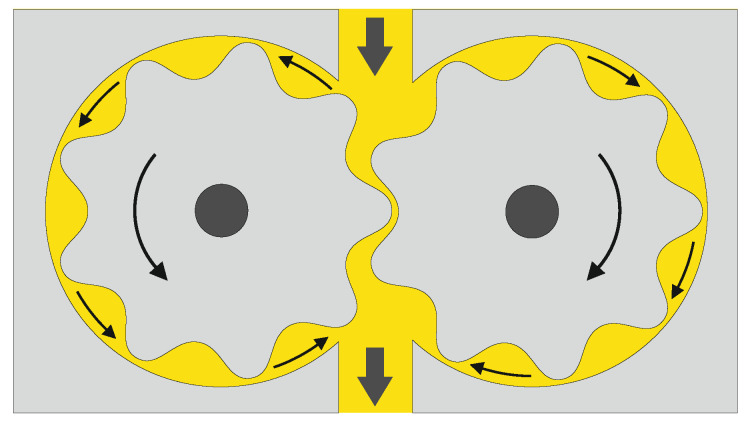
Working principle of a melt (gear) pump. By way of illustration, the polymer melt is represented in yellow.

The main function of the melt pump is to build up and maintain the required spin pressure, and to provide a controlled throughput of the melt [140]. Most common are gear pumps, where the rotating gears are filled from the extruder side and discharge the polymer on the downstream side (Figure 2). As long as the intermeshing gear teeth are completely filled by the melt, and tightly fit in the housing to minimize leakage flow, the discharge pressure stays virtually constant, independent of inlet pressure fluctuations [64]. However, the throughput is not only a function of capacity and rotation of the pump, but also of mechanical accuracy, pressure difference over the pump, and melt compressibility (at processing temperature and pressure). In this context, note that the density of a polymer increases with decreasing temperature and increasing pressure.

**Figure 3:**
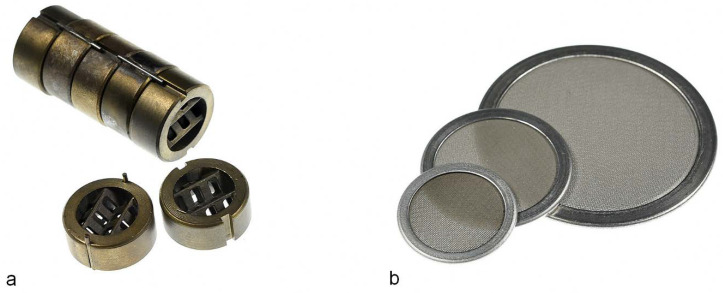
Examples of static mixers (**a**) and stainless steel filters (**b**).

A homogeneous melt is a must to achieve good processability and reproducibility, as well as decent mechanical properties of spun fibers. As mixing in a single-screw extruder is poor, static mixers (Figure 3 left) can be utilized to improve the distributive mixing and temperature uniformity of the melt stream [64,140]. Oerlikon Barmag (Remscheid, Germany) was the first to promote a gear pump with integrated rotating mixer for melt homogenization [141]. Filters made of steel screens, special sand or sintered metals remove foreign particles, gel particles and undesirable conglomerate additives, and thus prevent downstream equipment to be damaged by contaminants in the melt (Figure 3 right). Filters also build up additional melt pressure to improve melt quality and homogeneity due to their torturous path and high shear [142].

### 4.3. Spinneret

At the end of the extrusion line, the pressurized polymer melt is forced through the tiny holes (dies) of the spinneret to form continuous filaments. The design of the spinneret and the precision of the dies are crucial for good spinning results. A spinneret can consist of one up to several hundred fine holes which are quite sensitive to abrasion, corrosion and clogging by impurities (thus filtering the melt is of utmost importance). Filament uniformity and production yield strongly depend on die layout and length/diameter ratio (L/D) of the die [142], which has to be chosen taking the rheological behavior of a specific polymer into account.

**Figure 4:**
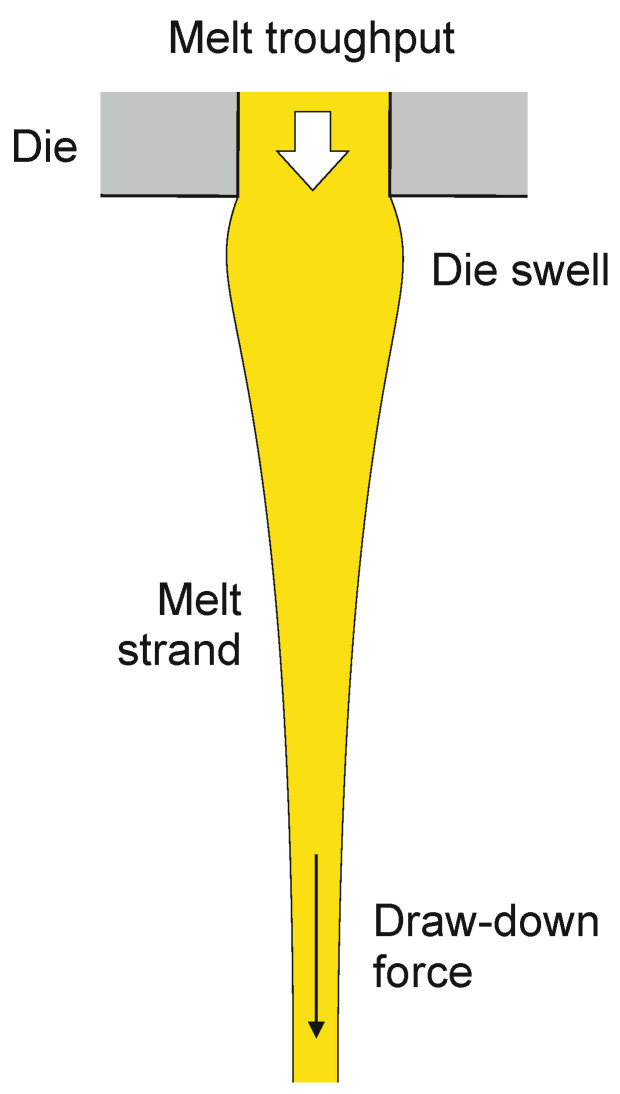
Die swell and fiber drawing. By way of illustration, the polymer melt is represented in yellow.

When passing through the spinneret, the polymer is subjected to shear flow that results in an increase of molecular orientation. When the melt emerges from the spinneret, the built-up elastic energy is released and the combined effects of surface tension and relaxation of molecular orientation result in die swell (Figure 4) [143]. When the draw-down force is too high, the melt might be drawn out of the die, hindering the development of die swell and leading to unsteady spinning conditions.

**Figure 5:**
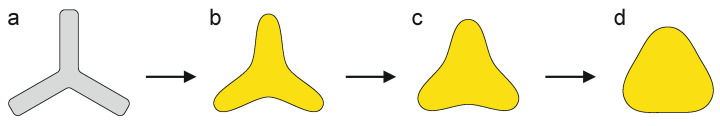
Typical die for trilobal cross-sections (**a**) and resulting trilobal filaments gaining in roundness due to increasing solidification time (**b–d**). By way of illustration, the polymer melt is represented in yellow.

Although the spinneret is mainly responsible for shaping the filament, designs of dies other than circular have to be handled with care. Trilobal cross-sections (Figure 5) are for example used in carpet yarns to achieve luster and higher stiffness, or for specialty filtration purposes [144]. Given enough time, the cross-section of the polymer melt exiting the die will adopt a circular shape due to surface tension and elasticity of the melt, independent of the capillary profile. Consequently, the original shape of the capillary can be retained the better, the faster the filament solidifies during quenching. Apart from surface tension, also die swell can contribute to a distortion of the cross-sectional profile.

### 4.4. Filament Draw-down Unit

**Figure 6:**
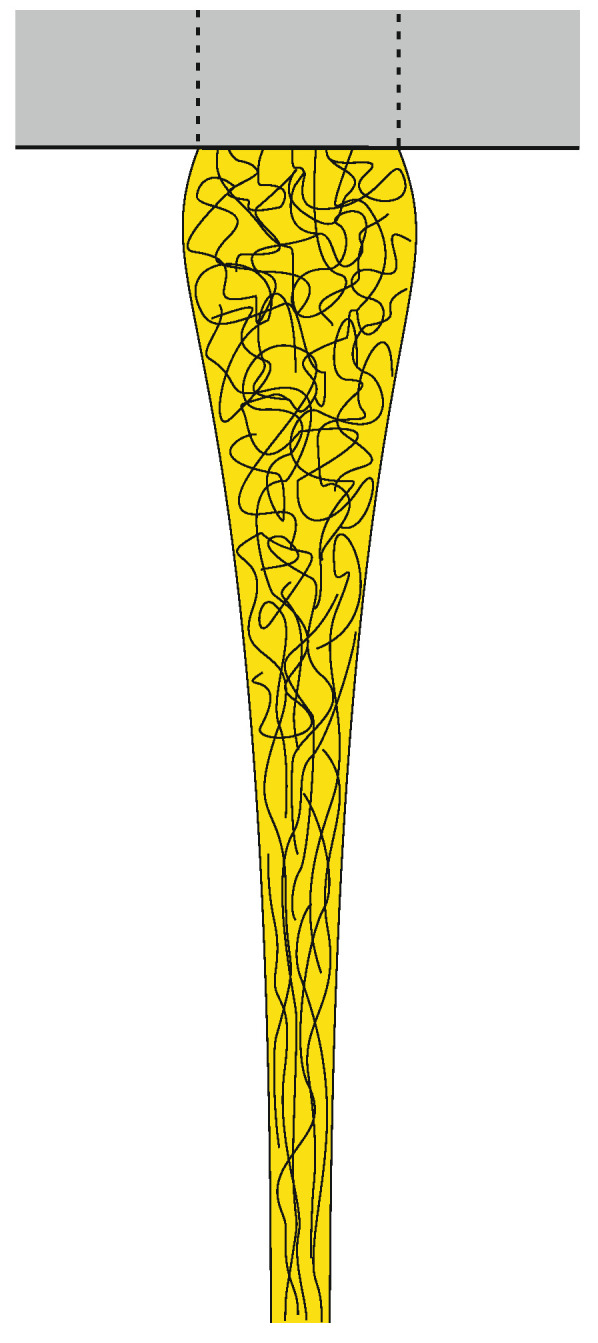
Orientation development along spinline in the case of high-speed spinning. By way of illustration, the polymer melt is represented in yellow.

The melt strand is commonly spun into a quenching chamber with adjustable air flow and quenching temperature. Thick filaments with diameters exceeding ~100 µm must be quenched in a water bath, because convective heat transfer by air is limited [145]. As the molten filament is accelerated down to the take-up roll, it is simultaneously cooled down and stretched uniaxial. In the case of high-speed spinning (PET: winding speed > 3500 m/min), the imposed stress is orienting the polymer chains and crystallizing the polymer (Figure 6) [143]. Depending on the type of polymer, orientation and crystallization might also develop at lower speed. The ratio between die exit velocity and take-up velocity is called draw-down ratio (DDR).

The degree of orientation in the filament depends on the extent of spin-line stress, which increases with decreasing melt temperature, with increasing molecular weight of the polymer, as well as with progressing quench and stretch rates [7,145]. However, too high viscosity and too fast cooling can subdue the drawability of the filament and prevent the necessary degree of deformation before the start of solidification [10]. Thus materials with long relaxation times and low spinnability are often spun into a post-heater (hot shroud) installed under the spinneret to improve stability, lower pre-orientation and reduce the solidification rate [7,146].

The melt-strength, which depends on the molecular weight of the polymer, must be sufficient to resist the forces of gravity, inertia and air or water friction [10]. Due to the high forces applied, a considerable viscous stretching can lead to a failure of filaments, i.e., surface tension-induced breakup into droplets, as the melt tends to minimize the surface area (Plateau-Rayleigh instability) [10,143].

The polymer has to solidify either through glass transition or crystallization before the filaments hit the take-up roll, otherwise they might stick to each other, on godets or winder. When the filaments solidify and travel downstream at high speed, the surface friction through air generates static electricity. A well-studied approach to improve the structural development of as-spun fibers is to guide them through a liquid isothermal bath during spinning [147,148,149,150]. By replacing the quenching air with a liquid, heat is transferred faster, and the increased frictional drag can impose higher spin-line stress [147].

To adjust frictional properties, prevent static charging and facilitate downstream processes, a spin finish (neat oil formulation or aqueous emulsion composed of lubricating oils, antistatic agents, emulsifiers, bactericides and other stabilizers) is used to lubricate the filaments [142]. The spin finish is applied just before the first godet by metering it onto the running filament with a gear pump.

Orientation of the macromolecules is mainly achieved by post-drawing in the solidified state by a set of godets operated at different revolving speeds [151]. Prime examples are polymers like PE, PP or PET, that can only be partially oriented in the fluid state [9]. During the drawing process, the as-spun filament is stretched up to several times its original length. The ratio between speeds of take-up roll and winder is called draw ratio (DR).

As high molecular mobility conditions favor homogeneous drawing, the respective temperature of slow crystallizing polymers (e.g., PET, PPS, PEEK) should be slightly above the glass transition temperature (T_g_), and a low crystallinity of the as-spun filament should be aimed for [152,153]. If as-spun filaments start to crystallize before drawing, they become brittle and undrawable [154]. Fast crystallizing polymers like PE and PP, on the other hand, usually already crystallize in an unoriented state before drawing is applied [155]. Here, molecular orientation develops in the crystallized stage, while slow crystallizing polymers orient in the non-crystalline state and develop oriented crystals during annealing (the latter drawn from the amorphous state, while polyolefins are drawn from the crystalline state) [55].

**Figure 7:**
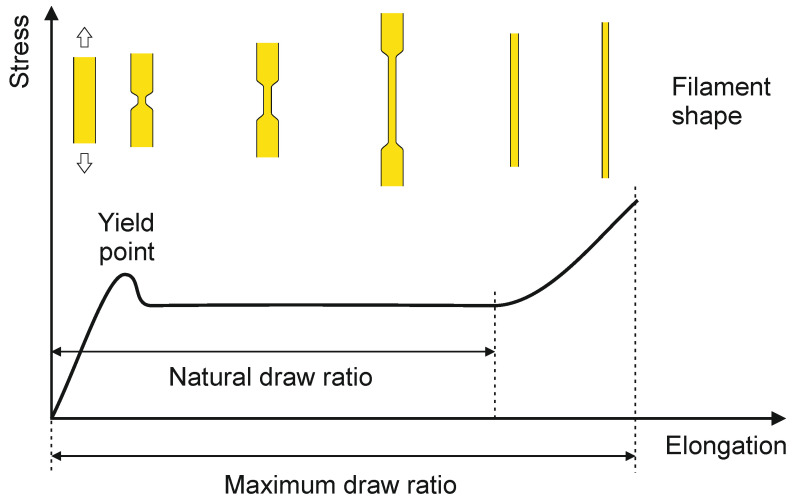
Stress-strain behavior of an as-spun filament.

When crystallizable polymers are subjected to high axial tension at a temperature below their melting point, they typically show necking (ductile failure), provided that the melt strength is high enough to prevent premature breakage [12]. This abrupt and drastic decrease in cross-sectional area generated in the filament by high local stress is characteristic to extension-thinning polymers [143,156], and is associated with stress-induced crystallization [37]. The reason a formed neck grows is that stress of force per unit area and thus extension rate are highest in the neck [157]. Operating below the natural draw ratio (Figure 7) can produce filaments with alternating necked and undrawn segments [154], which can be utilized to produce so-called “thick & thin” highly aesthetic fibers [158]. Depending on the polymer, excessive necking can be prevented by operating close to T_g_ (e.g., PET), by increasing drawing temperature, or by distributing the drawing process over several stages. To produce ultra-fine PET fibers, a flow drawing process is applied before regular drawing, since no necking occurs in flow drawing, and the development of orientation is minimal [159].

The morphological changes in neck-drawing of an already crystallized as-spun filament (e.g., PE) can be explained as follows (Figure 7) [9,143]:

- a strain imposed on a stack of crystal lamellae initially causes stretching of the interlamellar amorphous phases supporting the applied load (first, elastic part of the stress-strain curve);
- further stretching leads to slip-tilting and breakup of lamellae at the weakest points through chain pulling and unfolding, leading to the initial abrupt change in filament cross-section (yield point, indicates transition from elastic to plastic deformation);
- afterwards, as merely slippage of the lamellar fragments occurs, the filament shows deformation without much resistance and the required force remains practically constant (natural draw ratio);
- finally, all the chains are unfolded and the lamellar fragments, still connected by the pulled chains (tie molecules), form fibrils of alternating crystal blocks and stretched non-crystalline regions.

Drawing can be followed by annealing (heat setting) of the filaments under strain, to partially relax frozen-in stresses, and to perfect and stabilize the microstructure [145]. This reorganization is a result of increased chain mobility at elevated temperatures (i.e., between drawing temperature and T_m_). As a result, shrinkage, brittleness and tendency to fibrillate can be suppressed, and the filaments become soft and flexible. The transition from brittle to ductile behavior of fibers is a consequence of molecular reorientation, which can even be induced in the amorphous state. To facilitate handling of a multifilament in subsequent processes, intermingling of individual filaments can be applied by passing them through an air jet intermingler before they are wound up [155]. Man-made filaments can also be texturized to improve elasticity, tactility, bulkiness and insulation properties, to avert a glossy appearance, or to create special effects (fancy yarns) [160].

The main purpose of the winder is to provide a good package build (winding of filaments around and along bobbin) that permits high speeds in the downstream process, without impairing the filaments [160]. To achieve this, crossing and taper angles have to be optimized and filament tension between godets and winder has to be balanced. Post-shrinkage that can break fibers or crush the bobbin, or filament lengthening that would lead to a loose package, have to be prevented.

## 5. Physics of Melt-Spinning

### 5.1. Orientation in Polymer Fibers

In melt-spinning, drawing is the crucial step for extension and parallelization of macromolecules and crystallites along the filament axis, and thus achieving man-made fibers with a tensile strength about ten times higher than that of the as-spun filament [161,162]. Proper drawing results in row nucleated lamellae rather than spherulitic structures which would turn the fiber brittle [143]. In consequence, the physical properties of melt-spun fibers primarily derive from throughput rate (melt stream velocity) through the die, quenching rate (rate of cooling to solidify the filament) and take-up velocity (draw-down ratio) [142]. They are basically an indirect function of the molecular weight, as high molecular weight favors formation of highly oriented fibers. A main factor driving uniaxial orientation of the macromolecules is the stress at the solidification point, caused by air friction, mass inertia and rheological forces during filament formation [10]. With increasing strain rate, the polymer molecules start to disentangle and to align in the direction of flow [8]. Deformability and alignment of the polymer chains is mainly limited by their entanglements; less flexible, rigid-chain polymers (e.g., LCPs) repress chain entanglements and thus facilitate high molecular orientation [154].

As the filament cools in the spin-line, and entropic relaxation times exceed process (residence) times, imposed molecular orientation starts to persist [154]. Unless spinning speed is very high, flexible-chain polymers can only partially be oriented in the melt state, and their orientation has to be completed by post-drawing in the solidified state to build up their tensile properties [9]. To achieve further orientation of crystalline and non-crystalline regions, the as-spun filament is stretched between godets and winder (draw ratio). In semicrystalline polymers, drawing not only promotes orientation and morphology, but also rate and degree of crystallinity (crystallization induced by molecular deformation and orientation effects); this can lead to fibers with outstanding tenacities and moduli [7,163]. The crystallization rate during drawing is strongly affected by any previously introduced molecular orientation [142]. High orientation of macromolecules and crystallites results in a fibrillar structure; this explains why highly oriented fibers can be prone to fibrillation.

High take-up speeds result in high strain rates in the spin-line, giving rise to increased molecular orientation and strain-induced crystallization, and thus to reduced drawability of the as-spun filament [164]. In other words, improvements in tensile strength level off when the highest achievable orientation is reached. Delayed quenching by a post-heater (hot shroud) pushes the onset of solidification further in direction of the spin-line and thus reduces freeze-point stress [165]. As-spun fibers with a low degree of crystallinity are easier to draw, more extensive draw ratios are possible and higher tensile strength may be obtained [9]. As-spun filaments of slowly crystallizing polymers can remain nearly amorphous when a high cooling rate in the spin-line is applied [153].

### 5.2. Mechanical Properties of Melt-Spun Fibers

**Figure 8:**
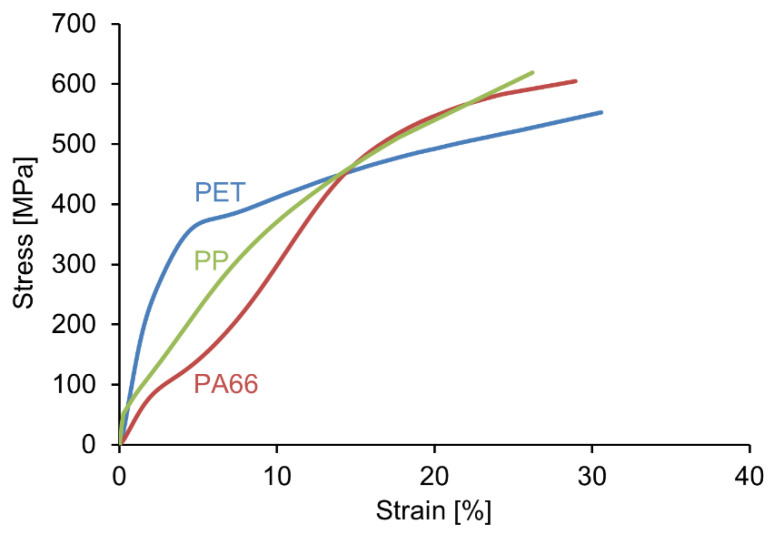
Typical stress-strain curves (own unpublished data) of common melt-spun monofilaments (PA 6.6, PP and PET, all diameter 80 µm).

Tensile properties of melt-spun fibers, in general, depend on molecular weight, molecular structure and orientation, glass transition temperature, and degree of crystallinity of the fiber [164]. Tensile properties of the most common melt-spun fibers, based on the linear flexible-chain polymers HDPE, PP, PET, PA 6 and PA 6.6, are in the range of 0.3–1.2 GPa tensile strength, 0.5–15 GPa Young’s modulus and 8–50% strain at break [166,167]. Fully drawn fibers, i.e., fibers drawn beyond their natural draw ratio (Figure 7), typically show load-strain curves as depicted in Figure 8.

Table 4 gives an example of physical properties of PET filaments produced in a one-step process, where the as-spun filament is directly taken up by a winder. In high-speed spinning (>3500 m/min), even slowly crystallizing polymers like PET undergo strain-induced crystallization. Excessive spinning speeds, however, can lead to reduced crystallinity (due to less crystallization time) and reduced molecular orientation, leading to poor mechanical performance of the resulting fibers [168].

### 5.3. Melt-Spinning Instabilities

Flow instabilities at best compromise performance and appearance of melt-spun filaments [143,146], and at worst completely impede melt-spinning. A typical instability affecting the flow of a polymer melt extruded through a capillary die is melt fracture, where a highly viscous extrudate exhibits a wavy distortion at high shear stress [170,171,172]. Occurrence of melt fracture depends on polymer type, average molecular weight and polydispersity, interface between polymer melt and die wall, capillary throughput (shear stress) and spinneret design. Melt fracture can be counteracted by increasing temperature, thus reducing viscosity, but with the risk of polymer degradation and loss in melt-strength.

**Figure 9:**
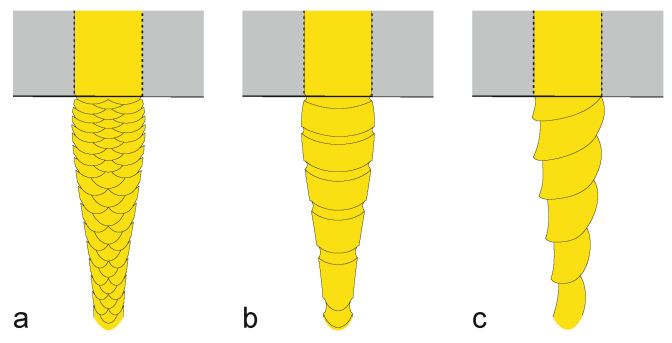
Illustration of the development of (**a**) sharkskin, (**b**) stick-slip (“bambooing”), and (**c**) gross melt fracture (“corkscrewing”). By way of illustration, the polymer melt is represented in yellow.

Spinning below a critical shear stress usually results in smooth fiber surfaces, but with increasing die throughput the surface can develop so-called sharkskin (Figure 9a), which is a periodic adhesive failure at the die exit due to dynamic crack penetration and subsequent healing [173]. Sharkskin is most commonly observed in highly entangled linear polymers with sufficiently narrow molecular weight distribution; amplitude and wavelength of this small periodic surface distortion increase with throughput rate [127]. Increasing the capillary diameter while maintaining the throughput reduces shear stress and thereby diminishes sharkskin formation.

Polymers that exhibit sharkskin typically also show a discontinuity known as stick-slip or oscillating melt fracture (“bambooing”, Figure 9b) when the shear stress exceeds a second critical value [173]. In contrast to sharkskin, the stick-slip instability is a volume distortion initiated in the die capillary, where flow rate and pressure oscillate, and the extrudate surface alternates periodically between relatively smooth (compression) and distorted (relaxation) portions [128,170]. Stick-slip corresponds to periodic transitions between weak and strong wall slip, as a result of sudden disentanglement of the polymer molecular chains in the bulk from chains attached to the capillary wall (cohesive failure), sustained by an interaction between compression of the melt in the reservoir upstream of the die and flow through the capillary [127,128,174,175,176]. Although the melt has only a small degree of compressibility, the large volume ratio between reservoir and capillary results in a pressure difference which is sufficient to sustain this oscillation [173]. The oscillation period increases with the volume of the reservoir and with the L/D ratio of the die, as longer capillaries result in higher pressures in the reservoir [128]. Such flow instabilities can be counteracted with processing aids like fluoropolymers that promote slippage [127].

At very high throughput so-called gross melt fracture can occur, which affects the entire cross-section of the extrudate, and originates from the acceleration of the pressurized melt in the contraction upstream of the die capillary [170,174,177,178]. A spiraling of the melt flow in the converging die entry can lead to helical extrudates (aka “corkscrewing”, Figure 9c) [172,174]. At even higher throughputs, the entrance instability becomes chaotic (aperiodic), involving true rapture and leading to a very rough filament [174]. All entangled polymer melts, also those not showing sharkskin or slip-stick, are limited in throughput by the development of gross melt fracture [173]. By streamlining the die inlet or by increasing the capillary length, the amplitude of the distortion can be reduced, but the critical shear stress for fracture is unchanged [172].

Another undesired spinning instability is the so-called draw resonance, which is a periodic undulation of the cross-section area that starts at a critical draw-down ratio where the mass flow rate between die exit and take-up godet is not constant anymore, although process conditions are being maintained constant [156,171,179]. To accommodate this mass flow variation at constant take-up speed, small fluctuations in the fiber cross-section produce oscillations in the filament tension, which, on their part, amplify the fluctuations [157]. Even when the rate of mass entering the region between die exit and take-up godet is constant, the rate of mass leaving it is not controlled, because only the take-up speed is regulated, and not the filament diameter [143]. In consequence, a thinning-out of one part of the melt strand is compensated by the thickening of another part, resulting in undulations of the cross-section. This self-sustaining resonance is more likely to develop when the filament is quenched in water near the spinneret to realize isothermal thinning.

The critical draw-down ratio depends on polymer properties, extrusion and spinning temperature, filament cross-section area, and the distance between spinneret and water bath [145]. Cooling and extension thickening have a stabilizing effect, and draw resonance can be prevented when a constant tension, rather than a constant take-up velocity, is applied to the filament [157].

## 6. Bicomponent Melt-Spinning

### 6.1. Objective of Bicomponent Spinning

Bicomponent melt-spinning offers the possibility to achieve polymeric filaments with extraordinary properties like ultra-fineness, light-guidance or electrical conductivity. Here, two polymers of different chemical and/or physical nature are extruded from one spinneret to form a single filament, with the goal to combine materials in elaborate fiber cross-sections [8]. The process of melt-spinning bicomponent filaments requires polymers with well-balanced processing temperatures and viscosities, and a good understanding of spin pack design and adhesion/bonding between the two components [180]. Although this process is technologically rather challenging, the range of bicomponent fibers and their applications has considerably grown since their launch in the 1960s [181].

The main objective of bicomponent melt-spinning is to exploit capabilities not existing in either polymer alone, as advantageous mechanical, physical or chemical properties of two materials can be combined in one fiber, expanding the range of possible applications [182,183]. As the two polymers can influence each other’s thinning and solidification behavior along the spinline, the molecular structure development of both components can be mutually affected [184]. Depending on the characteristics of the different polymers, bicomponent fibers are predominately commercialized as bonding elements in thermobonded nonwoven fabrics, as self-crimping fibers to achieve textured yarn, or as fibers with the surface functionality of special polymers and additives at reduced cost [142,185,186].

### 6.2. Cross-Section Geometries

**Figure 10:**
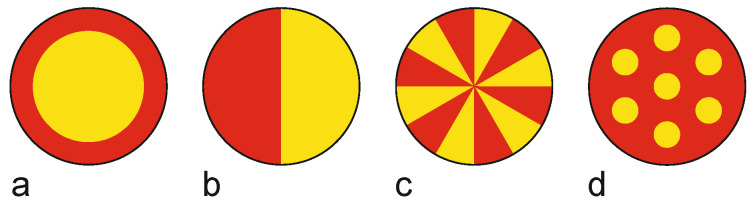
Typical cross-sections of bicomponent fibers: (**a**) core-sheath, (**b**) side-by-side, (**c**) segmented pie, (**d**) islands-in-the-sea. By way of illustration, the two fiber-forming polymers are represented in yellow and red, respectively.

The three main cross-section geometries of bicomponent fibers are core-sheath, side-by-side, and multiple cores configurations like segmented pie and islands-in-the-sea (Figure 10) [34]. As most thermoplastic polymers can be applied to clad a core which provides the requested tensile strength, the core-sheath approach enables a variety of surfaces while maintaining major fiber and textile properties [12]. Core-sheath types are commonly used as binder fibers for nonwovens, with a standard polymer as core and a low softening-point polymer as sheath [182,187]. When applied in nonwoven production, the core-sheath fibers are heated to a temperature high enough to cause the sheath to soften; consequently they will adhere to one another and stabilize the fabric [182].

Side-by-side bicomponent fibers are predominantly produced from two polymers that undergo differential shrinkage: after thermal treatment or relaxation, the filaments curl up and develop crimp contraction, which can be used to design self-crimping yarns applied in voluminous products [188,189]. Segmented pie and islands-in-the-sea configurations are mainly used to produce microfibers that are smaller in diameter than those obtained by conventional melt-spinning [190]. Segmented pie fibers are spun from two incompatible polymers that adhere poorly and split into microfibers when subjected to mechanical stress [142,191], while islands-in-the-sea fibers consist of microfibrils that are embedded in a dissolvable polymer matrix which will be removed in a follow-up process [12].

### 6.3. Spin Pack Design

When spinning bicomponent fibers, the polymers are extruded individually and kept separate up to the spin pack, where the melt flows meet and exit together through the spinneret to form a filament which consists of non-mixed components that touch at the interface [34]. When two polymer melts flow together, the interface dynamics considerably affect behavior and performance of the process, and all sorts of interface instabilities can occur [192].

**Figure 11:**
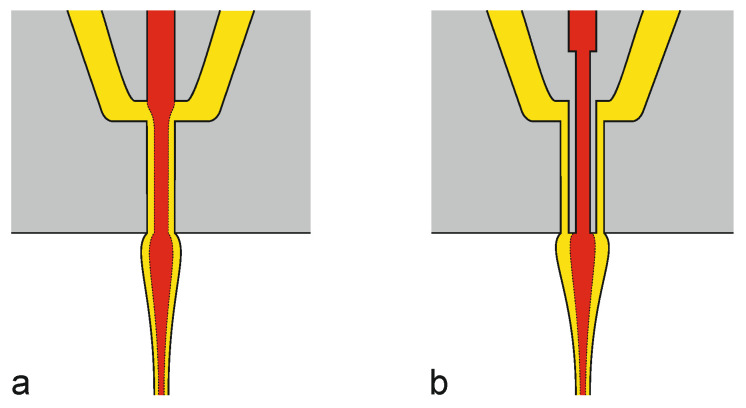
Two schematic examples of core-sheath bicomponent spinnerets: (**a**) single die and (**b**) multiple die. By way of illustration, the core and sheath polymer melts are represented in red and yellow, respectively.

Depending on the position where the molten polymers are supposed to merge, spinnerets for bicomponent fibers are classified as follows. In the by far most common single die type (Figure 11a), two pressurized polymer melts meet within the spinneret under laminar condition, which prevents mixing of the two flows [34]. Smaller spin-packs with shorter residence time, higher hole density and larger number of multiple cores can be realized by etching stacked plates to create the melt channels [144]. In the second, less prominent multiple die type (Figure 11b), the polymer melts meet just at the spinneret exit: this geometry is complex to realize, but it enables an exact positioning of the core polymer within the sheath, and it lessens rheological disparities between the two components [34].

## 7. Applications and Specialty Melt-Spun Fibers

### 7.1. Overview

For apparel use, melt-spun filaments are usually converted into threads resembling yarns made of natural fibers [160]. As a first step, a filament bundle is crimped and cut or torn into short fibers with lengths of several centimeters. These staple fibers are then converted into yarns by ring, open-end or air-jet spinning, and finally woven or knitted to get the desired textile fabrics.

For technical applications, man-made fibers are spun and used as multifilaments, which are superior in regularity and strength compared to staple fiber yarns. For special applications like fishing lines, brushes, artificial hair, hook-and-loop, filtration or silk-screen printing, monofilaments come into play. Due to their regularity and circular cross-sections, they are favored for precision fabrics.

High-performance melt-spun filaments are crucial for an immense number of technical applications. Table 5 summarizes categories of technical textiles, exemplary applications, and the most common polymers used to produce respective fibers [193]. Thanks to their cost-efficient production, nonwovens account for the highest tonnage of technical textiles [194]. An efficient alternative to produce nonwovens is to draw the filaments in the spinning process with high-speed air, and to deposit them randomly to form a fabric (spun-bond or melt-blown nonwoven).

### 7.2. Microfibers

Standard multifilaments range from 10 to 40 µm in diameter of the single filament, which in case of PET corresponds to a linear density of 0.1–1.7 tex (mg/m). A microfiber can be defined as a filament with a linear density below 0.1 tex. Finest directly melt-spun and drawn filaments go down to 5 µm (0.03 tex in case of PET). Ultra-fine fibers with diameters below 1 µm are produced in two steps: First, a multiple core bicomponent fiber is melt-spun, consisting of two incompatible polymers [34]. In a subsequent step, the two components are separated. In the case of a segmented pie configuration (Figure 10c), filaments of different polymers are split by mechanical means, while the sea (matrix) of island-in-the-sea fibers (Figure 10d) is dissolved to bare the island filaments. This second approach is ecologically disputable when solvents are needed, thus water-soluble matrix polymers are considered as sea material [195,196]. One of the first applications of ultra-fine fibers was synthetic leather called Alcantara^®^, followed by undergarments, sportswear and cleaning cloth. Owing to their high specific surface area, microfiber fabrics have excellent wicking and particle capture properties. Fabrics made from microfibers are very soft and flexible, inherently wind-proof thanks to their high fiber density, yet still easily permeable to water vapor (perspiration) [34].

### 7.3. Bioresorbable Fibers for Medical Applications

Resorbable fibers, mainly produced from biopolyesters like PGA, PLA, PCL and their copolymers, are prevailingly used as implants in the medical sector, with the advantage that they do not need to be removed by a second surgery [197]. Requirements are that the fibers and their degradation products show no toxic, inflammatory, immunogenic, carcinogenic or thrombogenic response, and that the fibers can be sterilized [198]. Main applications are surgical sutures, bone fixation devices, tissue engineering scaffolds, vascular grafts, temporary barriers and drug delivery devices [198]. The release rate of drug delivery fibers can be controlled by drug content, fiber diameter and polymer biodegradation [115]. Rate and degree of biodegradation depend on the polymer’s hydrophobicity, composition and degree of crystallinity; biopolymers degrade faster in the amorphous phase [77]. While PLA and PGA mainly degrade by chemical hydrolysis into acidic by-products, PCL and PHB are usually degraded by enzymatic hydrolysis [199,200]. PLA, PGA and their copolymers are widely used for tissue engineering applications; PCL and PHB, on the other hand, are more attractive for long-term implants, due to their hydrophobicity and low degradation rate [201].

### 7.4. Antimicrobial Fibers

An antimicrobial agent can either kill certain types of bacteria and fungi (biocide), or inhibit their growth [138]. In garments, antimicrobial agents can prevent unpleasant odors and staining caused by bacteria and fungi, which are fed by human sweat and sebum, as well as lubricants, auxiliaries and dirt [202]. Healthcare applications to protect customers against pathogenic microorganisms include hospital wear, wound care products and sterile bandages (traumatic, surgical and burn wound infections) [203]. In addition, industrial fabrics used for awnings, screens, tents, tarpaulins, ropes and similar products need protection from rotting and mildew as they are exposed to weather [202].

Some metals (silver, copper, zinc, gold, tin, mercury) and metal oxides (copper oxide, zinc oxide) show antimicrobial properties, preferentially in the form of nanoparticles, since nanosizing increases both reaction rate and contact area; their antimicrobial behavior is based on an oligodynamic effect, whose mechanism is not yet fully understood [138]. Silver nanoparticles (AgNPs) are the most effective broad-spectrum antibacterial agents, and respectively doped polymers found wide acceptability in medical and healthcare applications due to their excellent physicochemical and biological properties [204,205,206]. Parida et al. [207] found a solventless route to produce melt-spinnable AgNP-polymer composites by in-situ thermal reduction of silver precursors during extrusion.

### 7.5. Conductive Fibers

Conductive fibers can have a variety of functions, like antistatic protection, electromagnetic shielding and conductivity in electronic applications. Electrically conductive polymeric fibers are produced by dispersing conductive agents in polymer melts, by depositing carbon or metallic coatings onto fiber surfaces, or by incorporating hydrophilic comonomers [208]. Intrinsically conductive polymers (ICPs) exist, e.g., PAC, PPy, PANI, PTh, PPV, PPP, PF and PEDOT: PSS, and their conductivity can be tailored by doping via oxidation or reduction [209,210]. However, todays ICPs are not apt for melt-spinning due to poor stability, tenacity and processability, and reports on melt-spinning are limited to blends of conductive polymers with melt-spinnable polymers [209,211,212].

**Figure 12:**
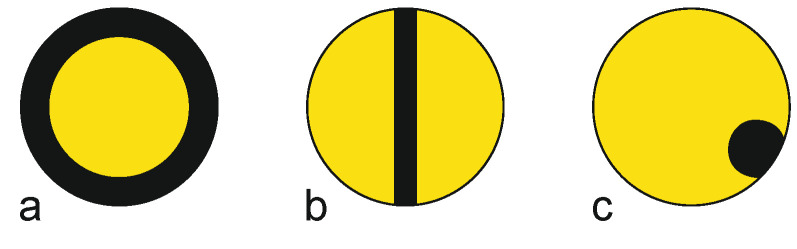
Exemplary cross-sections of antistatic bicomponent fibers: (**a**) concentric core-sheath, (**b**) sandwich side-by-side, (**c**) unequal side-by-side. By way of illustration, the pristine polymer is represented in yellow, and the black parts depict the antistatic component.

Antistatic fibers are required to prevent electrostatic charging by friction, thus preventing harmful discharge and adherence of particulates. In melt-spinning, the most convenient approach to overcome the intrinsic resistivity of man-made fibers is to add carbon black in high concentration to the matrix polymer. Since fibers with fillers have a reduced tenacity, antistatic fibers are usually melt-spun as bicomponent filaments to ensure sufficient tensile strength (Figure 12) [213].

### 7.6. Optical Fibers

Due to their mechanical flexibility and cost-efficiency, polymer optical fibers (POFs) are an attractive alternative to glass fibers [214]. An optical fiber transmits light along its axis by total internal reflection at the interface between a core and a sheath (cladding), where the refractive index of the core has to be higher than that of the cladding [215]. Common POF core materials are amorphous polymers like PMMA, PS, PCs or COPs, with PMMA having the lowest attenuation [66,69]. Based on their relatively low refractive index, fluoropolymers are typically utilized as cladding material [216].

A widespread POF manufacturing technique is heat-drawing from a preform, well-known from conventional glass fiber production [217,218]. While this process is discontinuous, bicomponent melt-spinning enables the continuous fabrication of POFs [34]. Sohn and Park [219] proposed graded-index POFs utilizing the diffusion of low molecular weight, high refractive index polymers in extruding PMMA bicomponent fibers with the help of a long capillary die. The relatively low processing temperatures even enable doping with luminescent dyes, which enables illuminating and light harvesting applications [220]. Despite their relatively high attenuation, POFs can be advantageous in textile applications where only short-distance light delivery, but high flexibility and good processability are required [216]. Apart from signal transmission and illumination, POFs also find applications in sensing, where the electronic equipment can be kept at a remote place free of electromagnetic noise [221].

### 7.7. Hollow and Liquid-Core Fibers

Hollow melt-spun fibers are mainly used in thermal insulation products [12], but they also have specialized applications in fluid separation [222,223]. To produce hollow fibers in a simple way, a spinneret with several separated arc slits is used, where the polymer melt flows through and merges at the die exit to form a tubular form [224,225,226]. However, with such spinnerets flow or weld lines cannot be prevented. In order to achieve a more uniform and robust fiber, a spinneret with annular co-flow channel can be applied, where the polymer melt is spun through the annulus, while a lumen fluid (air, nitrogen or CO_2_) is injected through the inner capillary [227,228].

**Figure 13:**
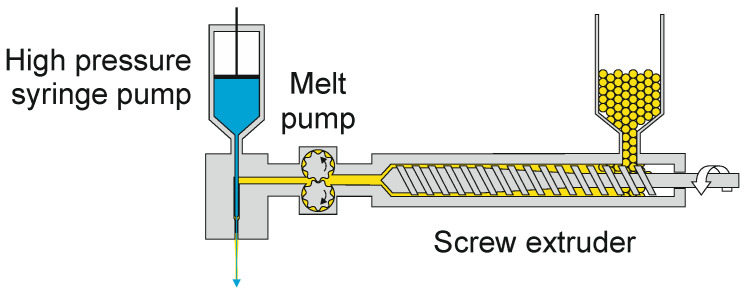
Schematic of liquid-core fiber production. By way of illustration, polymer and liquid are represented in yellow and blue, respectively.

Based on the same principle, Hufenus et al. [229] designed a special co-extrusion line that enables the pilot-scale production of liquid-filled polymeric fibers (Figure 13). The ability to produce a continuous liquid-core fiber is attractive since post-filling of a fine hollow filament would become increasingly slow and uneconomic at extended lengths [230]. Liquids in the core of a man-made filament can provide unprecedented properties regarding damping, flame retardancy, perfuming and many other applications, without compromising mechanical performance, dyeability or washability [231].

## 8. Concluding Remarks

The impressive success story of polymers has historically been kindled by the demand for new fibers. The development of filaments is strongly intertwined with research topics such as polymer synthesis and processing, multicomponent concepts, functionalization, medical and technical applications, or internet of things. The goal of melt-spinning is to create anisotropy in thermoplastic polymers, and the outcome are fibers that are high-performance, low-price, very durable and can be produced at large scale. Their quasi one-dimensional nature is the origin for a variety of applications, and the resulting textiles are both strong and flexible. However, due to the overall success of polymers, their mainly fossil-based sourcing, and humankind’s inappropriate disposal habits, man-made fibers are increasingly perceived as a global challenge in terms of resources, sustainability and waste management. Despite its utmost importance, this topic is beyond the scope of the present review. Nevertheless, continuous efforts to develop new types of sustainable polymers, fibers and textiles are definitely necessary for a prosperous future.

## Figures and Tables

**Table 1:** List of fiber spinning methods.

**Spinning Method**	**Material**	**Product**
melt-spinning	polymer or inorganic melt	filaments, staple fibers, textured yarns
wet-spinning, dry-spinning	polymer solution	filaments, staple fibers
gel-spinning	polymer gel (polymer and solvent)	filaments
preform drawing	polymer or inorganic melt	filaments
film-split spinning	polymer melt	slit-tape filaments
spun-bonding, melt-blowing	polymer melt	nonwovens
electrospinning, centrifuge spinning (force-spinning)	melt or solution of polymer or inorganic material	nonwovens
flash-spinning	polymer solution	nonwovens

**Table 2:** Typical properties of selected polymers used for melt-spinning [18,19,20,21,22,23,24,25,26,27]. Density, T_g_ and T_m_ are average values (exact data depend on degree of crystallinity and molecular weight). In the case of PLA, 98:2 L to D lactic acid is assumed [28]. The decomposition temperature T_d_ is defined by 5 wt% loss in N_2_, measured by thermogravimetric analysis (own unpublished data). Properties: TP = tensile properties, Res = resilience, ChR = chemical resistance, AR = abrasion resistance, UV = UV resistance, FR = flame retardancy. Performance: ++ = very good, + = acceptable, - = poor.

Polymer	Density [g/cm^3^]	T_g_ [°C]	T_m_ [°C]	T_d_ [°C]	TP	Res	ChR	AR	UV	FR
PA 6	1.14	50	225	387	++	++	+	++	+	+
PA 6.6	1.14	50	260	407	++	++	+	++	+	+
PET	1.39	75	260	402	++	+	+	+	+	+
PBT	1.33	50	220	373	++	++	+	+	+	+
PLA	1.25	60	165	321	+	+	+	-	+	+
PP	0.91	−15	170	399	++	-	++	+	-	-
LDPE	0.92	−125	110	440	+	-	++	-	-	-
HDPE	0.95	−125	130	436	++	-	++	+	-	-
PVDF	1.78	−40	170	431	-	++	++	-	++	++
PEEK	1.32	145	335	569	++	++	++	++	++	++
PPS	1.34	85	285	494	++	++	++	++	-	++
PEI	1.27	215	-	515	++	++	++	++	++	++
PMMA	1.18	110	-	334	-	+	-	+	++	-
PC	1.20	150	-	471	-	+	-	-	++	+

**Table 3:** Typical additives used in melt-spinning.

Type	Function	Examples
Processing aids	Antioxidant	Hindered phenols and amines, phosphites
	Hydrolysis stabilizer	Carbodiimide
	Nucleating agent	Talcum powder, boron nitride, organic phosphate salts
	Lubricant	Stearates, low molecular wax
	Polymer processing aid	Fluoropolymers
	Surfactant	Stearates, PEG
Enhancing additives	Plasticizer	Tributylcitrate, acetyltributylcitrate
	Chain extender	Difunctional acid derivatives, anhydrides and epoxides
	UV-stabilizer	HALS, TiO_2_, ZnO, carbon black
	Flame retardant	Phosphorous and halogen derivatives, HALS
	Thermal protection	Zirconia
Functional additives	Colorant	Pigments and dyes, carbon black
	Delustrant	TiO_2_, ZnO, mica, optical brightening agents
	Antistatic	Carbon black, carbon nanotubes, graphene, ZnO
	Antimicrobial	TiO_2_, ZnO, Ag^+^, Cu^2+^, Zn^2+^, plant extracts, phenol
	Water/oil repellent	Silicone and fluorine compounds

**Table 4:** Physical properties of PET filaments as a function of spinning speed (one-step process) [169].

Spinning Speed [m/min]	2000	4000	6000	8000
Ultimate tensile stress [MPa]	140–220	290–470	440–570	430–500
Ultimate tensile stress [cN/tex]	10–16	21–34	32–41	31–36
Ultimate tensile strain [%]	200–250	110–125	45–65	25–35
Young’s modulus [GPa]	2.1–2.8	3.5–6.1	8.2–9.5	11.5–12.8
Boiling water shrinkage [%]	58–62	20–57	3–5	2–3
Birefringence Δn	0.01	0.05	0.11	0.10–0.11
Degree of crystallinity [%]	2–11	4–27	40–48	41–50

**Table 5:** Technical textiles: Classification [193], applications and the three most common polymer classes for respective melt-spun fibers (indicated with +).

Application Field	Examples	Polyester	Polyamide	Polyolefin
Agrotech	Agriculture and horticulture (crop protection, fertilization), forestry, landscape gardening			+
Buildtech	Textile architecture (membrane construction), scaffolding	+		+
Clothtech	Functional garments and shoes	+	+	+
Geotech	Geotextiles (civil engineering, road & railroad construction)	+		+
Hometech	Domestic textiles (furnishing, carpets, drapery)	+	+	+
Indutech	Filtration, silk-screen printing, lifting, conveying, fishery	+	+	+
Medtech	Medical and hygiene products	+	+	+
Mobiltech	Automobiles (tire cord, belts, airbags, carpets, upholstery, insulation), railways, aircraft, ships	+	+	+
Oekotech	Recycling, waste disposal, environmental protection	+		+
Packtech	Packaging, carrier bags, ribbons	+		+
Protech	Protective clothing (safety workwear), property protection	+		+
Sporttech	Sports apparel, swimsuits, parachutes, climbing ropes, racket strings, artificial turf	+	+	

## Glossary

as-spun filament	filament spun and solidified without drawing & annealing
birefringence	measure of molecular orientation in polymers
bobbin (spool)	plastic or cardboard tube on which filaments are wound
die (nozzle)	orifice to eject a polymer solution or melt as coherent stream
die swell	relaxation and expansion of a polymer stream leaving the die
draw-down ratio (DDR)	ratio between die exit velocity and take-up velocity
draw ratio (DR)	ratio between speeds of take-up godet and winder
filament	continuous fiber, typically spun as multiple filaments
fully drawn yarn (FDY)	highly oriented filament(s) melt-spun with integrated drawing
glass-transition temperature (T_g_)	temperature where an amorphous material’s state changes from hard to viscous
godet (roller, galette)	revolving cylinder for heating and drawing of filaments
hopper (funnel)	tapered chamber to hold and dispense feed material (polymer granulate, chips)
low oriented yarn (LOY)	filament(s) melt-spun at low speed to be post-processed by drawing and texturizing
melting temperature (T_m_)	temperature where a crystalline material’s state changes from solid to liquid
necking	stress-induced, abrupt decrease in filament cross-sectional area, relevant for molecular orientation and crystallization
partially oriented yarn (POY)	partially oriented filament(s) to be post-processed by drawing & texturizing
spin finish	liquid applied to reduce electrostatic effects and facilitate processing of filaments
spinneret	equipment comprising multiple of dies to form filaments from a polymer solution or melt
staple fibers	filaments cut or torn into short fibers for yarn spinning or nonwoven production
texturizing	thermo-mechanical process to impart crimp and bulkiness to man-made filaments (draw textured yarn, DTY)
yarn count (titer)	linear density (mass per unit length) of fibers or yarns, with unit “tex” in the SI system (1 tex = 1 mg/m)
**Abbreviations**	
COP	cyclo olefin polymer
HDPE	high-density polyethylene
LCP	liquid-crystal polymer
LDPE	low-density polyethylene
P3HB	poly-3-hydroxybutyrate
P4HB	poly-4-hydroxybutyrate
PA 6	polyamide 6
PA 6.6	polyamide 6.6
PAC	polyacetylene
PANI	polyaniline
PBAT	polybutylene adipate terephthalate
PBT	polybutylene terephthalate
PC	polycarbonate
PCL	polycaprolactone
PEDOT:PSS	poly(3,4-ethylenedioxithiophene) polystyrene sulfonate
PEE	poly(ether ester)
PEEK	polyetheretherketone
PEF	polyethylene furanoate
PEI	polyetherimide
PEN	polyethylene naphthalate
PET	polyethylene terephthalate
PF	polyfluorene
PGA	polyglycolide
PHA	polyhydroxyalkanoate
PHBH	polyhydroxybutyrate-co-hydroxyhexanoate
PHBV	polyhydroxybutyrate-co-hydroxyvalerate
PLA	polylactic acid
PMMA	polymethylmethacrylate
PP	polypropylene
PPP	poly(p-phenylene)
PPS	polyphenylene sulfide
PPV	poly(p-phenylene vinylene)
PPy	polypyrrole
PS	polystyrene
PTFE	polytetrafluoroethylene
PTh	polythiophene
PTT	polytrimethylene terephthalate
PU	polyurethane
PVDF	polyvinylidene fluoride
PVF	polyvinyl fluoride
TPO	thermoplastic polyolefin elastomer
TPU	thermoplastic polyurethane
UHMWPE	ultra-high molecular weight polyethylene
